# A polarized high-field infrared instrument reveals giant infrared chirality in Weyl semimetals

**DOI:** 10.1093/nsr/nwag390

**Published:** 2026-06-25

**Authors:** Zhi-Ming Yu, Yugui Yao

**Affiliations:** Centre for Quantum Physics, Key Laboratory of Advanced Optoelectronic Quantum Architecture and Measurement (MOE), School of Physics, Beijing Institute of Technology, China; Beijing Key Lab of Nanophotonics and Ultrafine Optoelectronic Systems, Beijing Institute of Technology, China; International Center for Quantum Materials, Beijing Institute of Technology, China; Centre for Quantum Physics, Key Laboratory of Advanced Optoelectronic Quantum Architecture and Measurement (MOE), School of Physics, Beijing Institute of Technology, China; Beijing Key Lab of Nanophotonics and Ultrafine Optoelectronic Systems, Beijing Institute of Technology, China; International Center for Quantum Materials, Beijing Institute of Technology, China

## Abstract

This highlight introduces the work of Yuan et al., who observed the giant broadband infrared circular dichroism in the type-II Weyl semimetal Mn(Bi_1−x_Sb_x_)_2_Te_4_ for the first time through the development of a high-field magneto-infrared spectroscopy system with polarization resolution.

Chirality is a fundamental property of matter and profoundly shapes the optical and electronic responses of quantum materials. One of its most direct spectroscopic manifestations is circular dichroism, namely the differential absorption of left- and right-handed photons, which has enabled wide-ranging applications. However, in most natural materials, circular dichroism is intrinsically weak and confined to a narrow spectral range. Weyl semimetals have long been regarded as promising platforms for strong chiral light-matter interaction owing to their spin-momentum-locked electronic structure. Nevertheless, even for a single Weyl cone, electronic states at opposite momenta absorb photons of opposite helicity with equal strength, leading to the cancellation of circular dichroism at leading order.

**Figure 1.**
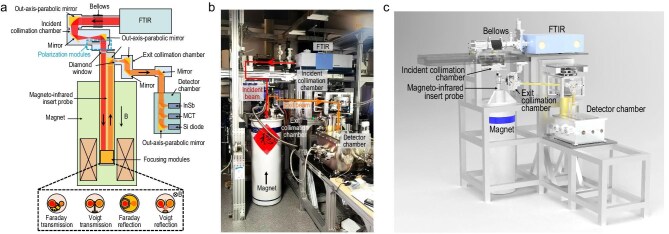
Schematic illustrations (a and c) and photograph (b) of the helicity-resolved magneto-infrared spectroscopy system [2]. Benefiting from a Kepler-type optical architecture, the polarizers and retarders can be integrated into the incident collimation chamber, enabling polarization-resolved magneto-infrared spectroscopy. Since the collimation chamber is located outside the magnet cryostat, switching between polarized and non-polarized configurations can be achieved without thermal cycling. Using this system, Xiang Yuan and collaborators discovered giant broadband circular dichroism in the type-II Weyl semimetal Mn(Bi_1-x_Sb_x_)_2_Te_4_. This pair of instrumental developments and scientific discoveries demonstrate an effective research pathway that progresses from scientific motivation to instrument construction, and from the instrumental construction to scientific discovery.

In our earlier theoretical work [1], we showed that strong magnetic fields can profoundly reconstruct the optical response of type-II Weyl fermions. The Landau levels in uncollapsed regime can host strong infrared absorption near Weyl nodes due to the overtilted band structure. When combined with the breaking of time-reversal symmetry, such enhanced infrared oscillator strength suggested a possible route toward strong circular dichroism in Weyl systems. This possibility, however, could only be tested by a broadband and polarization-resolved magneto-infrared spectroscopy instrument, which had not been available.

Yuan and co-workers now report both the required instrument and the resulting scientific discovery (Fig. 1) [2,3]. They developed a polarization-resolved high-field magneto-infrared spectroscopy system and used it to reveal giant and broadband infrared circular dichroism in the type-II Weyl semimetal Mn(Bi_1−x_Sb_x_)_2_Te_4_. This pair of instrumental and scientific works provides a particularly compelling example of a research mode in which an important scientific question drives instrument development, and the new instrument then enables the discovery of an otherwise inaccessible quantum-material response.

This long-sought instrumental advance has remained elusive in high-field Fourier-transform infrared spectroscopy, because the infrared beam must be delivered through meter-scale narrow light tubes into the magnet bore. Placing polarization optics near the sample can partly restore polarization purity, but it sacrifices broadband coverage, and *in-situ* polarization switching. Yuan and co-workers solved this long-standing challenge by introducing a collimated Kepler-type optical architecture constituted by incident and exit collimation chambers. The broadband infrared output is converted into a low-divergence beam, greatly reducing wall reflections. A remotely controlled polarizer-Fresnel-rhomb module outside the high-field cryogenic region, enables continuous tuning among linear, circular and elliptical polarization states without thermal cycling.

With this capability in hand, the first target was Mn(Bi_1−x_Sb_x_)_2_Te_4_. Under strong magnetic fields, this material is predicted to enter a ferromagnetic type-II Weyl phase [4,5]. The decisive ingredient is extreme particle-hole symmetry breaking, which reshapes the Landau-level wavefunctions and removes the circular dichroism cancellation. Helicity-resolved magneto-infrared spectroscopy not only demonstrates the strong intraband absorption at low frequencies that we predicted a decade ago, but also shows that interband Landau-level transitions couple almost exclusively to one circular-polarization channel, while the opposite channel is strongly suppressed. The giant circular dichroism reaches up to 130 mdeg/nm, and covers the 6–13 μm spectral range. It elevates particle-hole symmetry from a usually overlooked band-structure detail to an active control parameter.

The instrumental progress moves high-field infrared spectroscopy beyond the conventional measurement of frequency and intensity, activating polarization as a controllable experimental degree of freedom under extreme conditions. At the same time, the scientific discovery establishes a new and important route for disentangling symmetry, topology and chiral light-matter interaction in quantum matter.
